# Large hepatic biloma after bland hepatic arterial embolization using antireflux catheter

**DOI:** 10.1016/j.radcr.2022.12.045

**Published:** 2023-01-13

**Authors:** Richard Pham, Austin R. Swisher, Bassam Theodory, Jonathan Kessler

**Affiliations:** a University of California Riverside School of Medicine, 1550 Central Ave, Riverside, CA 92507, USA; b City of Hope Comprehensive Cancer Center, Duarte, CA, USA

**Keywords:** Biloma, Arterial embolization, Antireflux catheter

## Abstract

To our knowledge, there have been no previous reports of biliary injury or bilomas due to microvalve infusion catheters (Trinav Infusion System; Westminster, CO). Here, we describe an interesting case of large hepatic biloma following bland hepatic arterial embolization with an antireflux catheter.

## Introduction

Transarterial embolotherapy is part of the standard treatment algorithm for liver dominant neuroendocrine liver metastases. The microvalve infusion (MVI) catheters may offer several advantages over the standard end-hole (EH) catheters typically used in embolic procedures. By regulating pressure during infusions and preventing reflux to healthy tissue, MVI catheters may offer enhanced drug delivery with decreased adverse events. Biliary injury or biloma, however, is an uncommon complication after embolotherapy. Here, we discuss a case of hepatic biloma complication following arterial embolization with an anti-reflux catheter and its associated implications.

## Case presentation

**Fig. 1:**
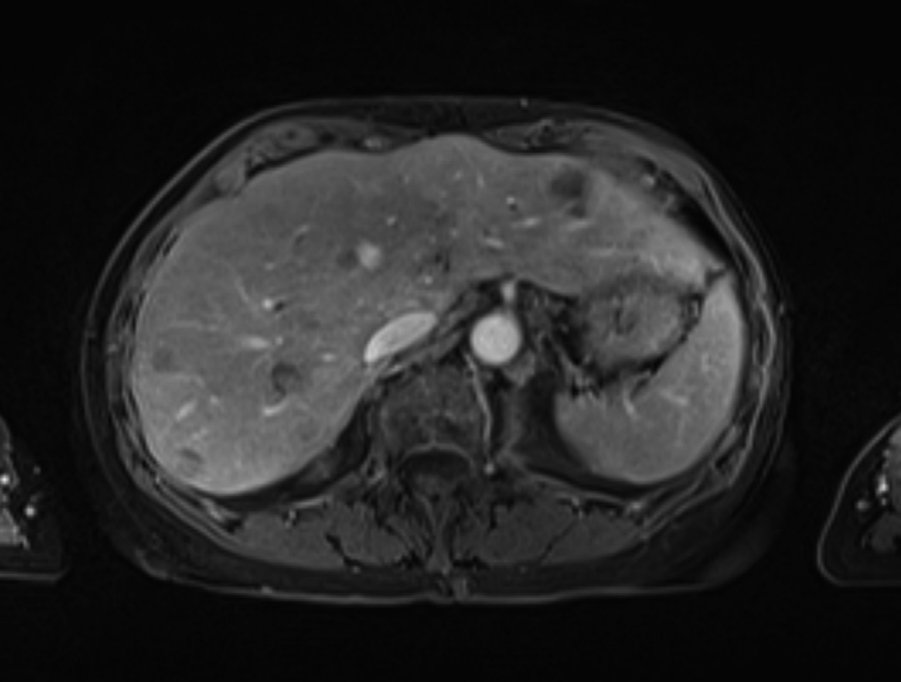
Pretreatment axial T1 postcontrast venous phase image of the liver with liver metastases.

**Fig. 2:**
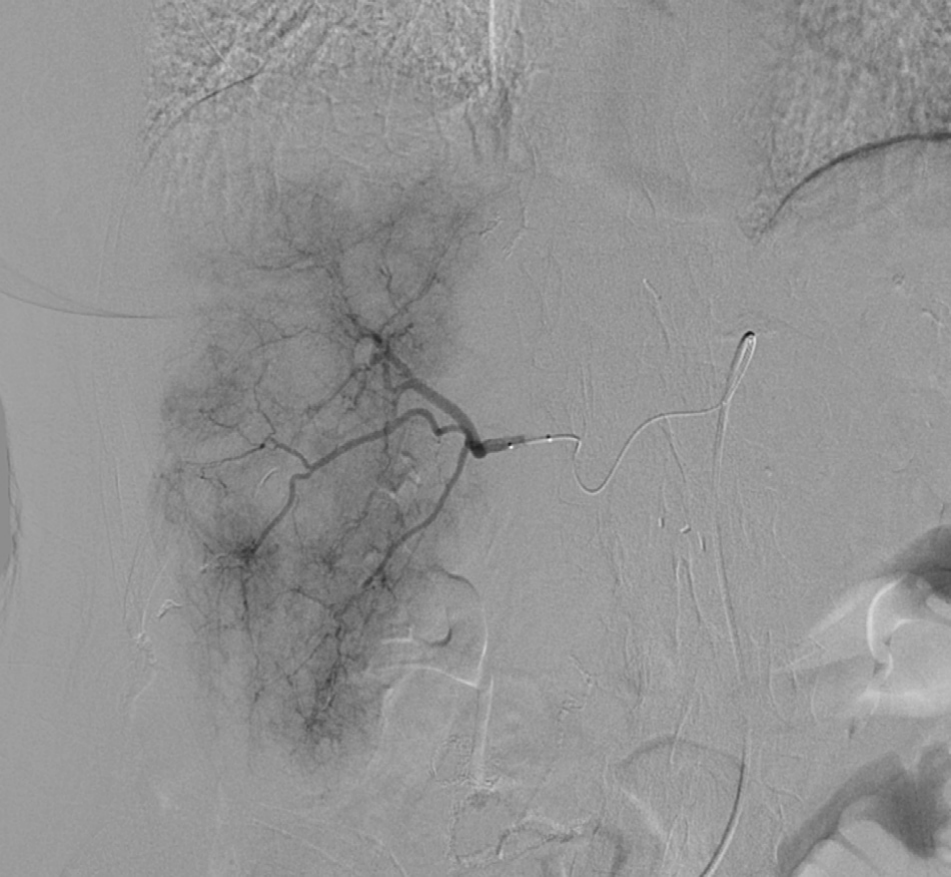
Superselective angiography through microvalve infusion catheter prior to particle embolization.

**Fig. 3:**
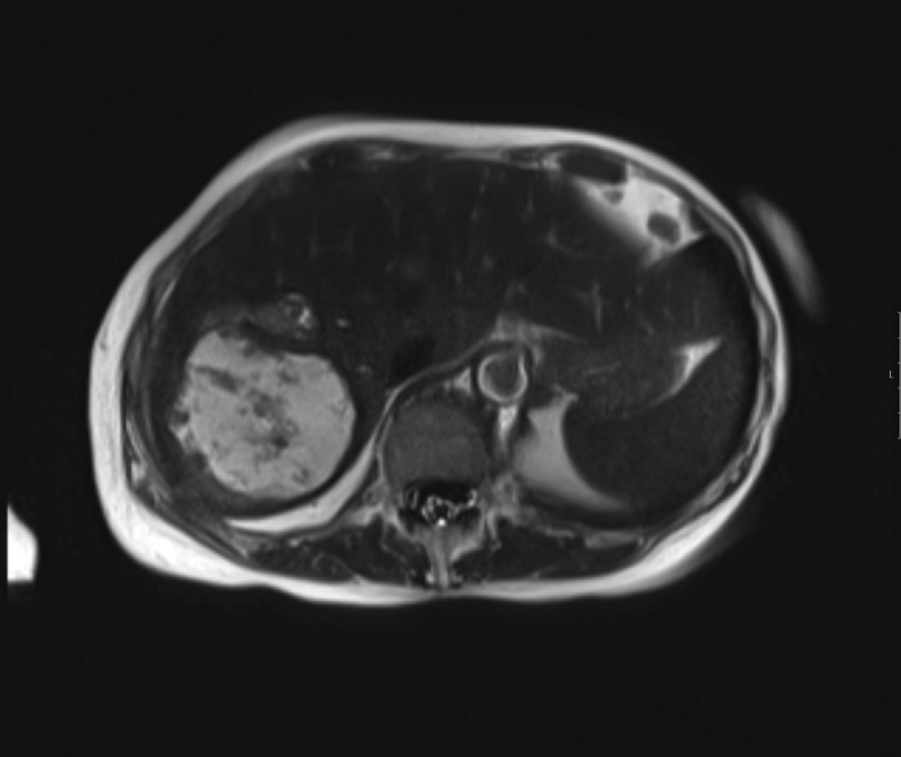
T2W magnetic resonance imaging of the liver demonstrating high signal fluid collection within the treated area of the liver.

A 70-year-old female presented with a history of well-differentiated neuroendocrine tumor of the small bowel with metastasis to the peritoneum and lymph nodes. The patient initially underwent surgical debulking followed by treatment with long-acting somatostatin analogs for approximately 3 years. She subsequently presented with new, progressive liver metastases. She initially underwent bland embolization to the lateral left lobe of the liver using 100 micron particles (Embozene; Palo Alto, CA) through a standard 2.8 Fr EH catheter. She was discharged on post-op day 1 and recovered without complication. Approximately 1 month later, patient imaging demonstrated a response in the treated liver and continued progression in the untreated right liver. She subsequently returned for planned bland embolization of the tumor in the right lobe of the liver. Representative pretreatment imaging was performed for comparison (Fig. 1). Embolization was performed at this time with a MVI catheter (MVI) (Trinav Infusion System; Westminster, CO) 1 vial of 100 micron particles (Embozene; Palo Alto, CA) (Fig. 2). She was discharged on post-op day 1 with mild postembolic symptoms. She then presented again 3 weeks later with persistent symptoms of fatigue, decreased appetite, abdominal, and pelvic discomfort. Liver function tests were normal, with the exception of elevated alkaline phosphatase (230 U/L; normal range 20-140 U/L). Computed tomography with contrast demonstrated a large, multiloculated cystic lesion in the right hepatic lobe consistent with a biloma. Magnetic resonance imaging confirmed a complex cystic lesion in the right hepatic lobe measuring 8.3 × 6.6 × 4.5 cm (Fig. 3). The patient was treated conservatively with analgesics. Repeat imaging performed three months later demonstrated stable hepatic metastasis and improvement in the intrahepatic biloma. Her symptoms were completely resolved by 3 months without further intervention.

## Discussion

Traditionally, transarterial embolotherapy has been performed with either conventional chemoembolization (cTACE) utilizing a chemotherapy-lipiodol emulsion, bland embolization utilizing microparticles, or radioembolization utilizing Yttrium-90 microspheres. To date, no studies have demonstrated clear supremacy of any specific embolic agent. Biliary injury or biloma is a relatively uncommon finding after embolotherapy [1]. This is thought to occur due to ischemia of the peribiliary plexus. While the liver parenchyma has a dual blood supply from the hepatic artery and hepatic portal vein, the isolated arterial supply to the intrahepatic bile ducts makes them particularly susceptible to ischemia. This appears to occur more commonly in patients with neuroendocrine tumors and in those treated with drug-eluting bead TACE [2]. This is hypothesized to occur due to a relatively underdeveloped peribiliary plexus in patients with neuroendocrine tumors compared to those with hepatocellular carcinoma who more commonly have cirrhosis and compensatory hypertrophy of the biliary plexus [2].

MVI catheters have numerous benefits in comparison to standard EH catheters. With an expandable tip acting as a 1-way valve, MVI catheters allow users to target tumors with more precision and avoid the associated reflux [3]. Additionally, MVI catheters may preferentially direct blood flow into tumors through changes in flow dynamics [4]. By reducing downstream pressure from the MVI catheter, compensatory vasoconstriction may occur in the healthy parenchyma's vascular supply. Tumor arterioles, however, are less likely to vasoconstrict due to abnormal innervation, smooth muscle, and autoregulatory properties; this may result in increased uptake of drug or particles within the tumor vasculature and uniform drug distribution within tumors, and tumor necrosis [4]. Similarly, through these proposed mechanisms, MVI catheters are thought to enhance distal penetration and decrease reflux flow to healthy, neighboring liver parenchyma.

Reports of MVI catheters have also demonstrated similar rates of complications and hepatotoxicity when compared to EH catheters [3,4]. To our knowledge, however, there have been no previous reports of biliary injury or bilomas due to MVI catheters. We hypothesize that the use of the MVI catheter increased the exposure of liver parenchyma and bile ducts to ischemic injury by relatively increasing the distal embolic particle delivery and may have increased the likelihood of this occurrence. We therefore suggest judicious use of MVI catheters in patients with metastatic disease particularly when employing small caliber bland particle embolization. Further investigation is needed to identify which patients may be at increased risk for complications.

## Compliance with ethical standards

Written informed consent was obtained from the patient for the publication of this case report.

## Ethics approval

The information provided for the above submission was evaluated and determined by an institutional review board to not meet the definition of human subject research. Accordingly, IRB approval and continuing review is not required.

## Patient consent

Written and informed consent was obtained for publication from the patient.
